# Supplementary material for: Assessment of Cu(II) Removal from Aqueous Solutions by Modified Pomelo Peels: Experiments and Modelling
Source: Molecules. 2023 Apr 13;28(8):3438. doi: 10.3390/molecules28083438 (PMC10145579; doi:10.3390/molecules28083438)
Supplement: Supplementary file 1 [file molecules-28-03438-s001.zip › molecules-2337873-supplementary.pdf]

Supplementary Data

Assessment of Cu(II) Removal from Aqueous Solution by Modified Pomelo Peels: Experiments and Modelling

Ruixue Zhang[1], Mengqing Jiao[1], Nan Zhao[1,2*], Johan Jacquemin[3], Yinqin Zhang[4], Honglai Liu[2]

[1] Hebei Province Key Laboratory of Sustained Utilization & Development of Water Resources, Hebei GEO University, Shijiazhuang 050031, China

[2] School of Chemistry and Molecular Engineering, East China University of Science and Technology, Shanghai 200237, China

[3] Materials Science and Nano-Engineering MSN Department, Mohammed VI Polytechnic University, Lot 660-Hay Moulay Rachid, Ben Guerir 43150, Morocco

[4] School of Water Conservancy and Hydroelectric Power, Hebei University of Engineering, Handan 056038, China

Content:

The experimental data of the Cu(II) adsorption by MPP collected in this work are tabulated in Table S1. The training data used for the ANN model during this work are tabulated in Table S2. The testing data used for the ANN model during this work are tabulated in Table S3.

Corresponding Author: Nan Zhao, E-mail: zhaonan@hgu.edu.cn

Table S1. Experimental Data of the Cu(II) Adsorption by MPP Measured during this Work

No.	pH	*T* (K)	C_o (mg/L)	t (min)	C_e (mg/L)	q_e (mg/g)	*R* (%)
1	2	298.15	20	60	2.7930	4.3018	86.04%
2	3	298.15	20	60	1.3548	4.6613	93.23%
3	4	298.15	20	60	1.2502	4.6875	93.75%
4	5	298.15	20	60	1.1129	4.7218	94.44%
5	6	298.15	20	60	1.4104	4.6474	92.95%
6	7	298.15	20	60	2.0799	4.4800	89.60%
7	5	298.15	20	10	3.4139	4.1465	82.93%
8	5	298.15	20	20	1.7096	4.5726	91.45%
9	5	298.15	20	30	1.3678	4.6581	93.16%
10	5	298.15	20	40	1.2390	4.6903	93.81%
11	5	298.15	20	50	1.1747	4.7063	94.13%
12	5	298.15	20	60	1.1129	4.7218	94.44%
13	5	298.15	4	60	0.9098	0.7726	77.26%
14	5	298.15	8	60	0.9918	1.7521	87.60%
15	5	298.15	12	60	1.0228	2.7443	91.48%
16	5	298.15	16	60	1.0929	3.7268	93.17%
17	5	298.15	20	60	1.1129	4.7218	94.44%
18	5	298.15	24	60	1.1546	5.7114	95.19%
19	5	298.15	28	60	1.2025	6.6994	95.71%
20	5	288.15	20	60	1.4336	4.6416	92.83%
21	5	293.15	20	60	1.1712	4.7072	94.14%
22	5	298.15	20	60	1.1129	4.7218	94.44%
23	5	303.15	20	60	1.0301	4.7425	94.85%
24	5	308.15	20	60	0.9633	4.7592	95.18%
25	5	313.15	20	60	0.9267	4.7683	95.37%

Table S2 Training Data used for Developing the ANN model during this Work

No.	pH	*T* (K)	C_o (mg/L)	t (min)	C_e (mg/L)	q_e (mg/g)	*R* (%)
1	2.0	298.15	20	60	2.7930	4.3018	86.04%
2	2.2	298.15	20	60	2.3079	4.4230	88.46%
3	2.6	298.15	20	60	1.6641	4.5840	91.68%
4	2.8	298.15	20	60	1.4752	4.6312	92.62%
5	3.0	298.15	20	60	1.3548	4.6613	93.23%
6	3.2	298.15	20	60	1.2877	4.6781	93.56%
7	3.6	298.15	20	60	1.2530	4.6868	93.74%
8	3.8	298.15	20	60	1.2552	4.6862	93.72%
9	4.0	298.15	20	60	1.2502	4.6875	93.75%
10	4.2	298.15	20	60	1.2271	4.6932	93.86%
11	4.6	298.15	20	60	1.1543	4.7114	94.23%
12	4.8	298.15	20	60	1.1246	4.7188	94.38%
13	5.0	298.15	20	60	1.1129	4.7218	94.44%
14	5.2	298.15	20	60	1.1270	4.7183	94.37%
15	5.6	298.15	20	60	1.2284	4.6929	93.86%
16	5.8	298.15	20	60	1.3106	4.6723	93.45%
17	6.0	298.15	20	60	1.4104	4.6474	92.95%
18	6.2	298.15	20	60	1.5250	4.6188	92.38%
19	6.6	298.15	20	60	1.7883	4.5529	91.06%
20	6.8	298.15	20	60	1.9319	4.5170	90.34%
21	7.0	298.15	20	60	2.0799	4.4800	89.60%
22	5.0	298.15	20	10	3.4139	4.1465	82.93%
23	5.0	298.15	20	12	2.8963	4.2759	85.52%
24	5.0	298.15	20	16	2.1488	4.4628	89.26%
25	5.0	298.15	20	18	1.8963	4.5259	90.52%
26	5.0	298.15	20	20	1.7096	4.5726	91.45%
27	5.0	298.15	20	22	1.5773	4.6057	92.11%
28	5.0	298.15	20	26	1.4312	4.6422	92.84%
29	5.0	298.15	20	28	1.3948	4.6513	93.03%
30	5.0	298.15	20	30	1.3678	4.6581	93.16%
31	5.0	298.15	20	32	1.3408	4.6648	93.30%
32	5.0	298.15	20	36	1.2863	4.6784	93.57%

No.	pH	*T* (K)	C_o (mg/L)	t (min)	C_e (mg/L)	q_e (mg/g)	*R* (%)
33	5.0	298.15	20	38	1.2611	4.6847	93.69%
34	5.0	298.15	20	40	1.2390	4.6903	93.81%
35	5.0	298.15	20	42	1.2209	4.6948	93.90%
36	5.0	298.15	20	46	1.1942	4.7015	94.03%
37	5.0	298.15	20	48	1.1840	4.7040	94.08%
38	5.0	298.15	20	50	1.1747	4.7063	94.13%
39	5.0	298.15	20	52	1.1655	4.7086	94.17%
40	5.0	298.15	20	56	1.1440	4.7140	94.28%
41	5.0	298.15	20	58	1.1301	4.7175	94.35%
42	5.0	298.15	4	60	0.9098	0.7726	77.26%
43	5.0	298.15	5	60	0.9432	1.0142	81.14%
44	5.0	298.15	7	60	0.9819	1.5045	85.97%
45	5.0	298.15	8	60	0.9918	1.7521	87.60%
46	5.0	298.15	9	60	0.9984	2.0004	88.91%
47	5.0	298.15	10	60	1.0042	2.2490	89.96%
48	5.0	298.15	12	60	1.0228	2.7443	91.48%
49	5.0	298.15	13	60	1.0394	2.9902	92.00%
50	5.0	298.15	14	60	1.0588	3.2353	92.44%
51	5.0	298.15	15	60	1.0778	3.4806	92.81%
52	5.0	298.15	17	60	1.1018	3.9746	93.52%
53	5.0	298.15	18	60	1.1063	4.2234	93.85%
54	5.0	298.15	19	60	1.1090	4.4727	94.16%
55	5.0	298.15	22	60	1.1296	5.2176	94.87%
56	5.0	298.15	23	60	1.1414	5.4646	95.04%
57	5.0	298.15	24	60	1.1546	5.7114	95.19%
58	5.0	298.15	25	60	1.1680	5.9580	95.33%
59	5.0	298.15	27	60	1.1928	6.4518	95.58%
60	5.0	298.15	28	60	1.2025	6.6994	95.71%
61	5.0	288.15	20	60	1.4336	4.6416	92.83%
62	5.0	289.15	20	60	1.3503	4.6624	93.25%
63	5.0	291.15	20	60	1.2347	4.6913	93.83%
64	5.0	292.15	20	60	1.1976	4.7006	94.01%
65	5.0	293.15	20	60	1.1712	4.7072	94.14%
66	5.0	294.15	20	60	1.1528	4.7118	94.24%

**No.**	**pH**	***T* (K)**	**C_o (mg/L)**	**t (min)**	**C_e (mg/L)**	**q_e (mg/g)**	***R* (%)**
67	5.0	296.15	20	60	1.1310	4.7172	94.34%
68	5.0	297.15	20	60	1.1227	4.7193	94.39%
69	5.0	299.15	20	60	1.0997	4.7251	94.50%
70	5.0	301.15	20	60	1.0661	4.7335	94.67%
71	5.0	302.15	20	60	1.0478	4.7381	94.76%
72	5.0	303.15	20	60	1.0301	4.7425	94.85%
73	5.0	304.15	20	60	1.0138	4.7466	94.93%
74	5.0	306.15	20	60	0.9858	4.7536	95.07%
75	5.0	307.15	20	60	0.9739	4.7565	95.13%
76	5.0	308.15	20	60	0.9633	4.7592	95.18%
77	5.0	309.15	20	60	0.9539	4.7615	95.23%
78	5.0	311.15	20	60	0.9384	4.7654	95.31%
79	5.0	312.15	20	60	0.9321	4.7670	95.34%
80	5.0	313.15	20	60	0.9267	4.7683	95.37%

Table S3. Testing Data used for the ANN Model Validation.

No.	pH	T (K)	C_o (mg/L)	t (min)	C_e (mg/L)	q_e (mg/g)	R (%)
1	2.4	298.15	20	60	1.9366	4.5158	90.32%
2	3.4	298.15	20	60	1.2588	4.6853	93.71%
3	4.4	298.15	20	60	1.1918	4.7021	94.04%
4	5.4	298.15	20	60	1.1663	4.7084	94.17%
5	6.4	298.15	20	60	1.6518	4.5871	91.74%
6	5.0	298.15	20	14	2.4784	4.3804	87.61%
7	5.0	298.15	20	24	1.4883	4.6279	92.56%
8	5.0	298.15	20	34	1.3133	4.6717	93.43%
9	5.0	298.15	20	44	1.2062	4.6985	93.97%
10	5.0	298.15	20	54	1.1556	4.7111	94.22%
11	5.0	298.15	6	60	0.9665	1.2584	83.89%
12	5.0	298.15	11	60	1.0116	2.4971	90.80%
13	5.0	298.15	16	60	1.0929	3.7268	93.17%
14	5.0	298.15	21	60	1.1197	4.9701	94.67%
15	5.0	298.15	26	60	1.1811	6.2047	95.46%
16	5.0	290.15	20	60	1.2848	4.6788	93.58%
17	5.0	295.15	20	60	1.1403	4.7149	94.30%
18	5.0	300.15	20	60	1.0837	4.7291	94.58%
19	5.0	305.15	20	60	0.9991	4.7502	95.00%
20	5.0	310.15	20	60	0.9457	4.7636	95.27%
